# Supplementary material for: FAM13A affects body fat distribution and adipocyte function
Source: Nat Commun. 2020 Mar 19;11:1465. doi: 10.1038/s41467-020-15291-z (PMC7081215; doi:10.1038/s41467-020-15291-z)
Supplement: Supplementary file 1 — Supplementary Information [file 41467_2020_15291_MOESM1_ESM.pdf]

Supplementary Information

FAM13A affects body fat distribution and adipocyte function

Fathzadeh *et al*.

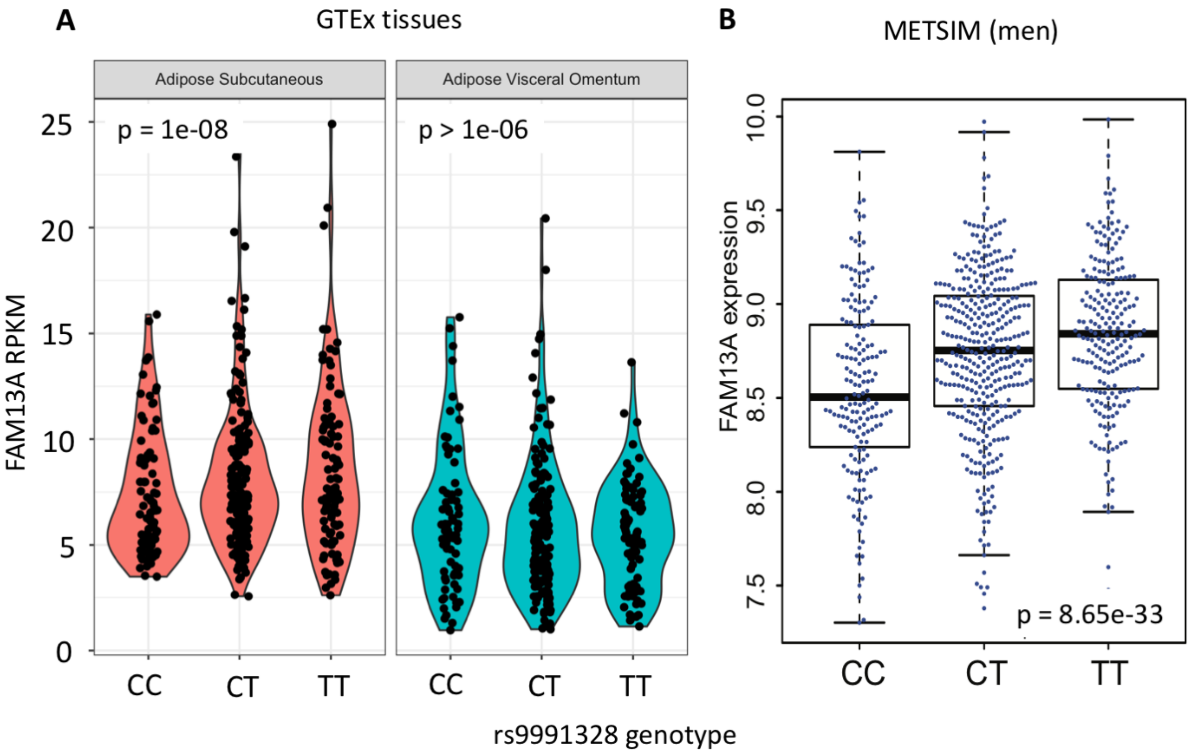

Supplementary Figure 1. Adipose eQTLs variants within *FAM13A* locus. The T allele of the leading variant from Bayesian finemapping analysis of the GTEx SAT association signal for *FAM13A* expression (rs9991328) is associated with increased SAT FAM13A expression in GTEx (A) and METSIM (B) cohorts. The association signal is much weaker in GTEx VAT (A).

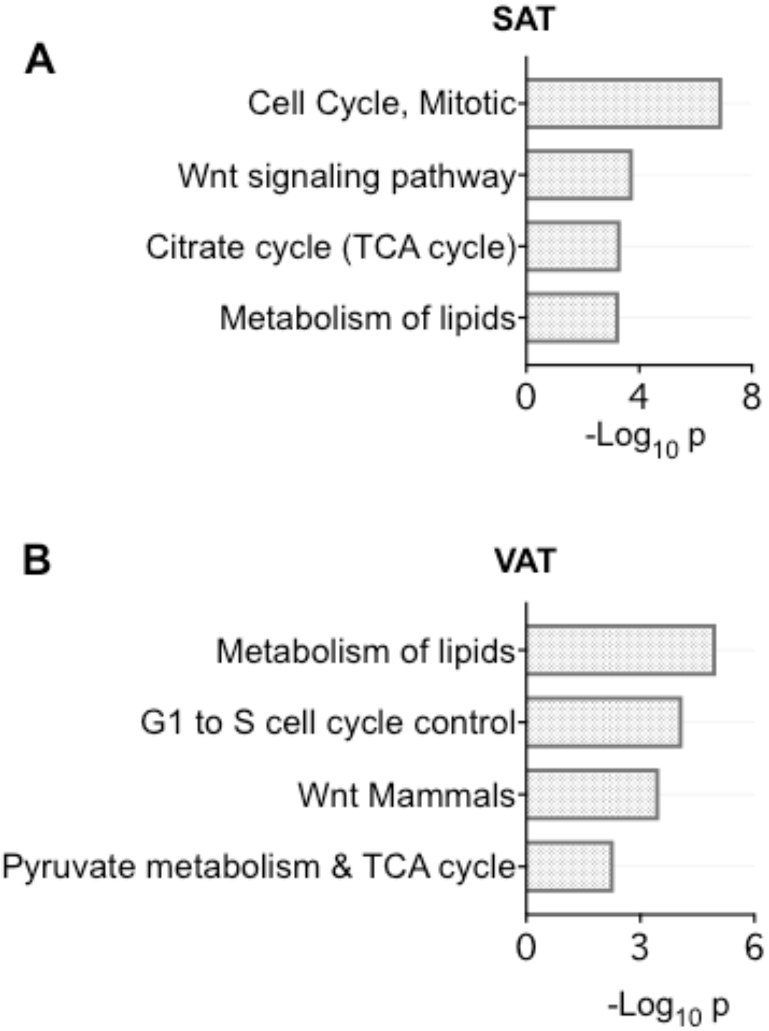

Supplementary Figure 2. Pathway analysis of adipose *FAM13A*-correlated geneset. Over-representation analysis (using ConsensusPathDB[15]), **A**) in men SAT (METSIM expression data) and **B**) men and women VAT depots (STAGE expression data). For full list of significant pathways see Supplementary Excel file.

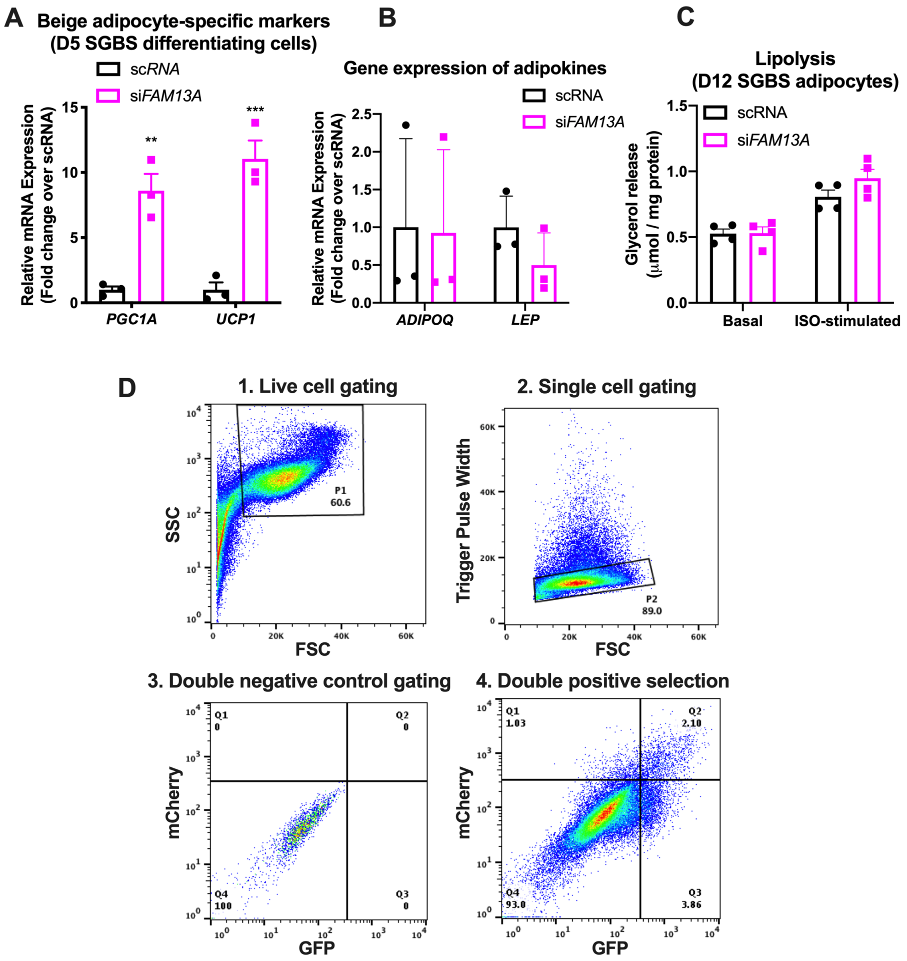

Supplementary Figure 3. Effects of *FAM13A* on human SGBS adipocyte functions beyond adipogenesis and establishment of CRISPRi perturbation of *FAM13A* in SGBS cells. **A)** mRNA expression of beige adipocyte markers (*PGC1A, UCP1*), measured 5 days after adipogenic induction in cells transfected with scrambled siRNA or si*FAM13A*. **B)** mRNA expression of white adipocyte markers (*ADIPOQ, LEP)*, measured on D10 adipocytes and 2 days after siRNA transfection. **C)** Basal and isoproterenol-stimulated lipolysis measured in the conditional medium of D12 differentiated adipocytes and 4 days after siRNA transfection. Data are presented as mean±SEM. n=3 independent experiments. *p<0.05, ***p<0.001. **D)** Representative FACS gating strategy used to obtain SGBS preadipocytes expressing CRISPRi machinery and the corresponding gRNAs (detailed in Methods): 1. Cells were initially selected by size, on the basis of forward scatter (FSC) and side scatter (SSC); 2. Cells were then gated on both FSC and SSC singlets to ensure that individual cells were analyzed; 3. Non-infected SGBS preadipocytes were used to determine background fluorescence levels; 4. Representative FACS collection gates used to sort SGBS preadipocytes infected with both dCas9-KRAB (mCherry positive) and sgRNAs (GFP positive). The abundance of the relevant cell subpopulation was labelled in the graph. (n=3 independent experiments).

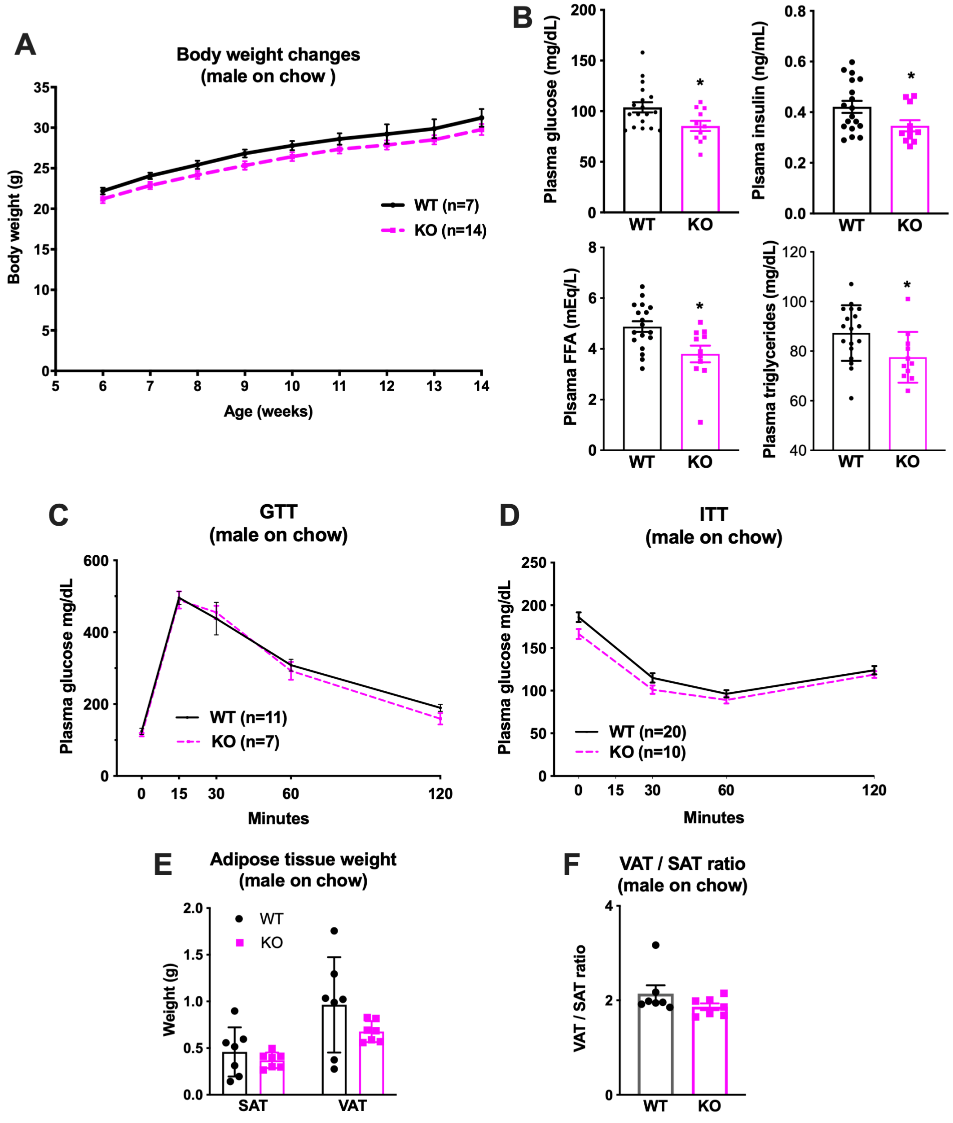

G

	SAT		VAT	
	WT (µm)	KO (µm)	WT (µm)	KO (µm)
Minimum	20.01	20	20.05	20.03
25% Percentile	27.99	27.53	33.6	37.845
**Median**	**36.585**	34.47	**46.29**	54.225
75% Percentile	47.4175	43.4775	63.3575	72.4325
Maximum	151.42	107.3	286.54	170.69

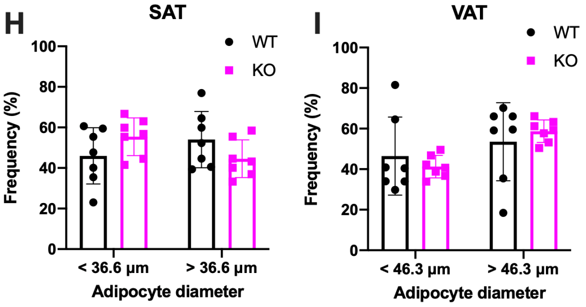

Supplementary Figure 4. Metabolic characterization of male *Fam13a* KO mice on chow diet. A) Body weight of male WT and *Fam13a* KO mice fed on chow from 6 weeks to 15 weeks old. (n=7 per WT, n=14 per KO). **B**) Serum lipid profiles (glucose, insulin, free fatty acids and triglycerides) in 15 weeks old of male WT and *Fam13a* KO mice fed on chow, measured after 12-hour fasting. (n=18 per WT, n=11 per

KO), * P < 0.05. **C-D**) Glucose and insulin tolerance tests on 15 weeks old of male WT and *Fam13a* KO mice fed on chow. Blood glucose levels were measured at baseline and after intraperitoneal (I.P.) injections of glucose (C) or insulin (D) at 15-, 30-, 60, 120-minute. (For GTT, n=11 per WT, n=7 per KO; for ITT, n=20 per WT, n=10 per KO). **E**) Tissue masses (g) of 14 weeks old of male WT and *Fam13a* KO mice fed on chow. (n=7 per group). **F)** Ratio of VAT/SAT, based on tissue masses, in 14 weeks old of male WT and *Fam13a* KO mice fed on chow. (n=7 per group). **G)** Percentile analysis of adipocyte sizes in SAT or VAT, based on H&E images (20x magnification) of VAT and SAT in 14 weeks old of male WT and *Fam13a* KO mice fed on chow. (n=7 per group). **H-I)** Percentage of cells smaller or larger than the median of WT cells (illustrated in **S4G**) in either SAT (**H**) or VAT (**I**) depot, in 14 weeks old of male WT and *Fam13a* KO mice fed on chow. (n=7 per group).

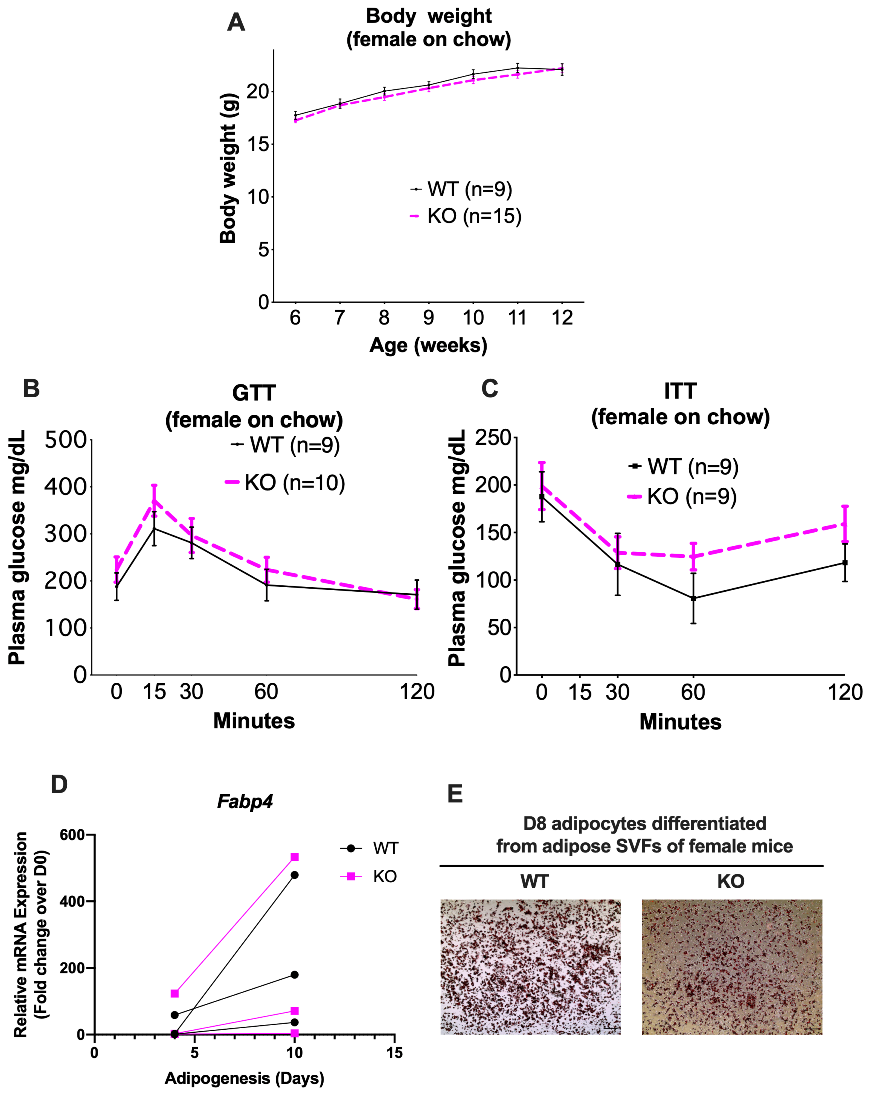

Supplementary Figure 5. Metabolic profiling of female *Fam13a* KO mice on chow diet. A) Body weight of female WT and *Fam13a* KO mice fed on chow from 6 weeks to 12 weeks old. (n=9 per WT, n=15 per KO). **B**-**C**) Glucose and insulin tolerance tests on 14 weeks old of female WT and *Fam13a* KO mice fed on chow. Blood glucose levels were measured at baseline and after intraperitoneal (I.P.) injections of glucose (B) or insulin (C) at 15-, 30-, 60, 120-minute. (For GTT, n=9 per WT, n=10 per KO; for ITT, n=9 per WT, n=9 per KO). **D**) mRNA expression of adipogenic markers (*Fabp4*), measured by qRT-PCR during *in vitro* adipogenesis of cultured SVFs (D0, D4, D8) isolated from SAT of female mice (n=3 per group, n=3 culture wells per animal). **E)** Oil-Red O staining of lipid droplets in D8 differentiated adipocytes from SVFs. (n=3 per group, pictures represent n=3 independent experiments, n=3 cultures/animal, 4x magnification, scale bar=250 μm).

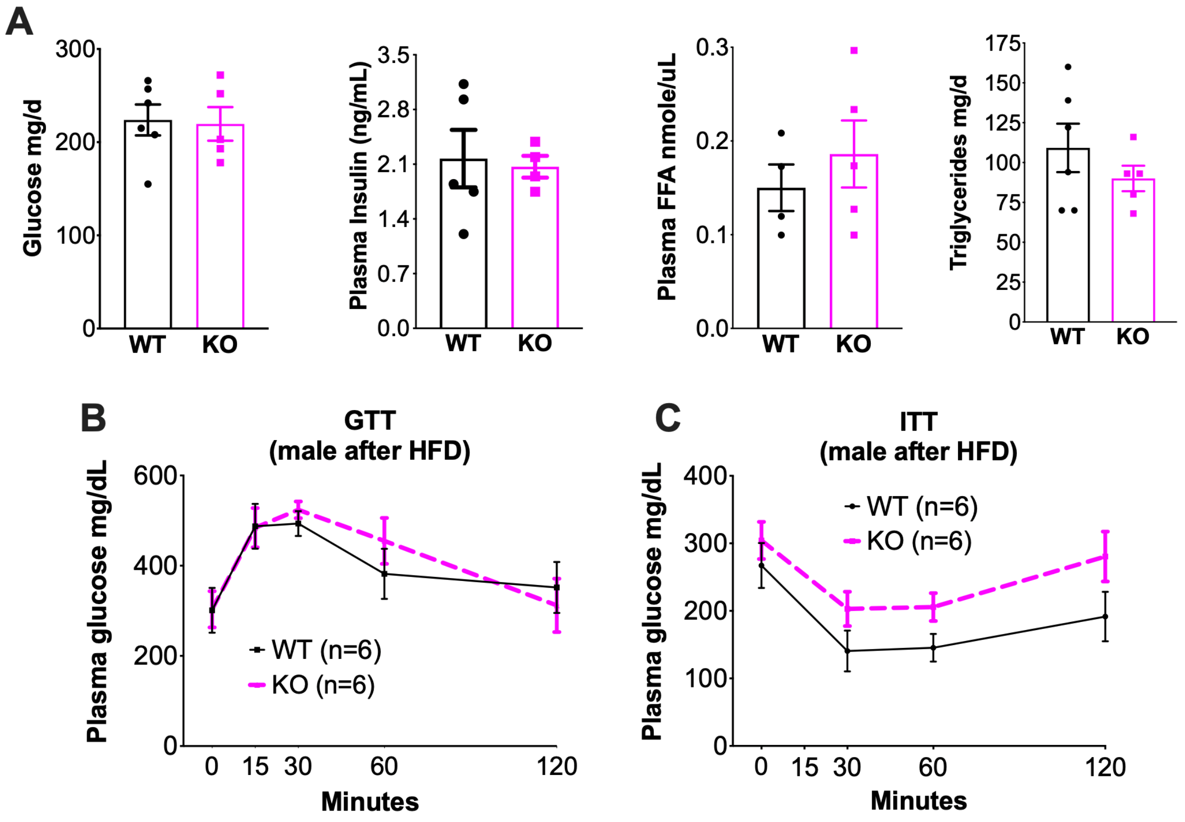

Supplementary Figure 6. Metabolic profiling of male *Fam13a* KO mice on high fat diet. A) Serum lipid profiles (glucose, insulin, free fatty acids and triglycerides) in male WT and *Fam13a* KO mice after 14 weeks HFD, measured after 12-hour fasting. (n=6 per group).**B-C**) Glucose and insulin tolerance tests on male WT and *Fam13a* KO mice after 14 weeks HFD. Blood glucose levels were measured at baseline and after intraperitoneal (I.P.) injections of glucose (C) or insulin (D) at 15-, 30-, 60, 120-minute. (n=6 per group). All values are presented as mean±SEM. *p<0.05.

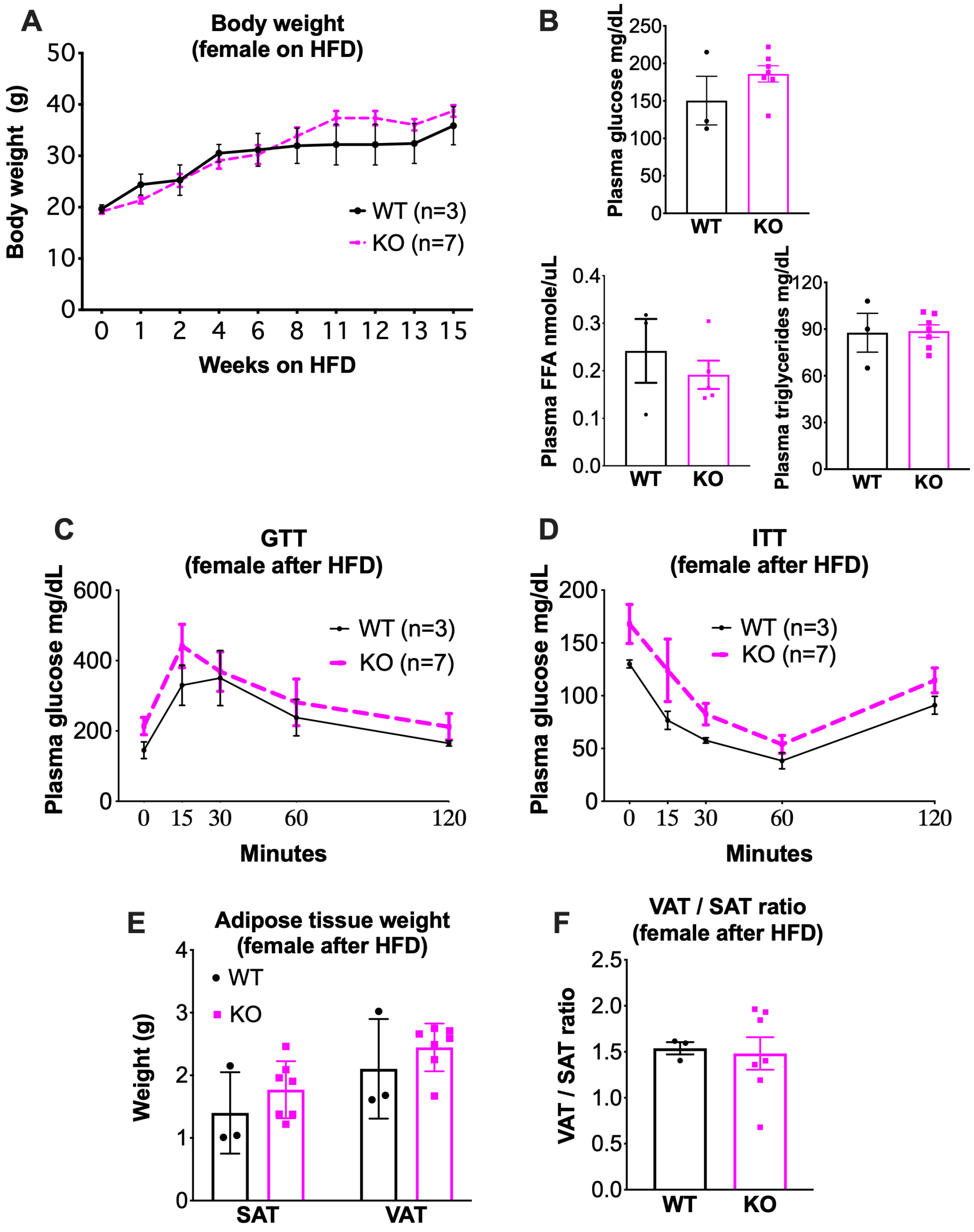

Supplementary Figure 7. Metabolic profiling of female *Fam13a* KO mice on high fat diet. A) Body weight of female WT and *Fam13a* KO mice fed on HFD for 14 weeks, from 8-week to 22-week old. **B**) Serum lipid profiles (glucose, insulin, free fatty acids and triglycerides) in female WT and *Fam13a* KO mice after 14 weeks HFD, measured after 12-hour fasting. **C-D**) Glucose and insulin tolerance tests on female WT and *Fam13a* KO mice after 14 weeks HFD. Blood glucose levels were measured at baseline and after intraperitoneal (I.P.) injections of glucose (C) or insulin (D) at 15-, 30-, 60, 120-minute. **E**) Tissue masses (g) of female WT and *Fam13a* KO mice after 14 weeks HFD. **F)** Ratio of VAT/SAT, based on tissue masses, in female WT and *Fam13a* KO mice after 14 weeks HFD. (n=3 per WT group, n=7 per KO group). All values are presented as mean±SEM.

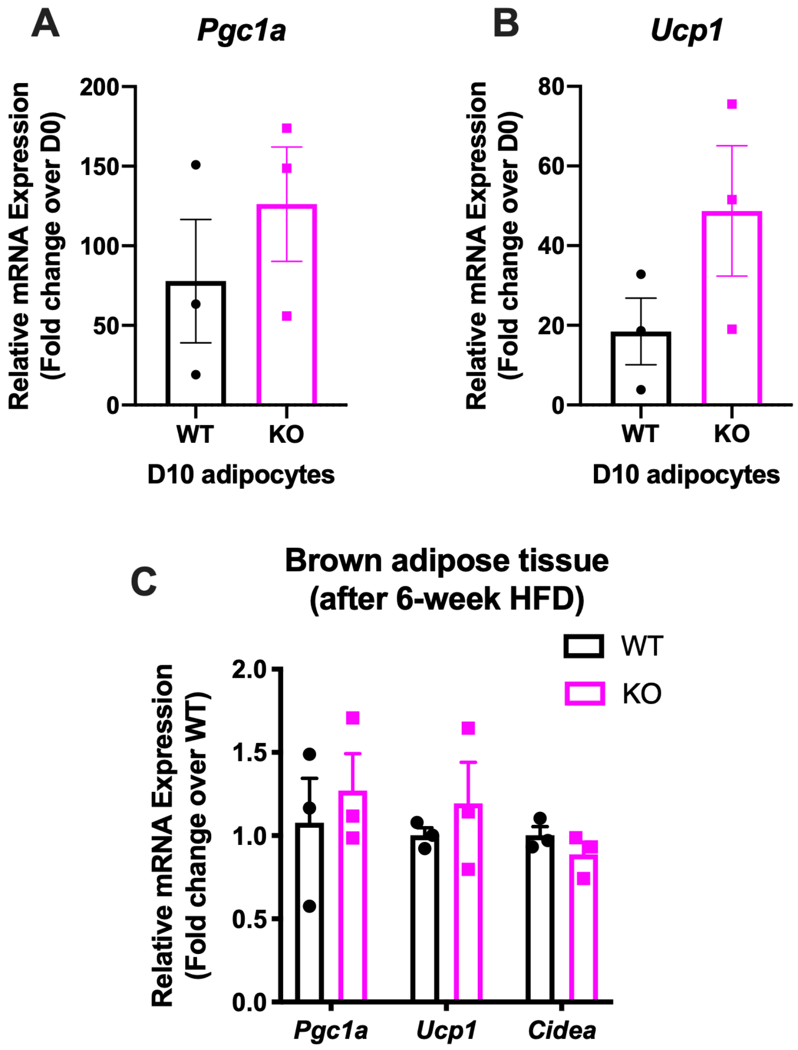

Supplementary Figure 8. Effects of *Fam13a* on expression of brown/beige markers in mouse beige adipocytes or brown adipose tissue. A-B) mRNA expression of beige markers, *Pgc1a* (A) and *Ucp1* (B), measured by qRT-PCR during *in vitro* adipogenesis of cultured SVFs (D0, D4, D10). (8-week-old male WT or *Fam13a* KO mice when isolating SVFs, n=6 per group, n=3 culture wells per animal). **C)** mRNA expression of brown markers (*Pgc1a*, *Ucp1, Cidea)*, measured by qRT-PCR in RNA extractions of brown adipose tissue (BAT) after 6-week HFD. (n=3 per group).

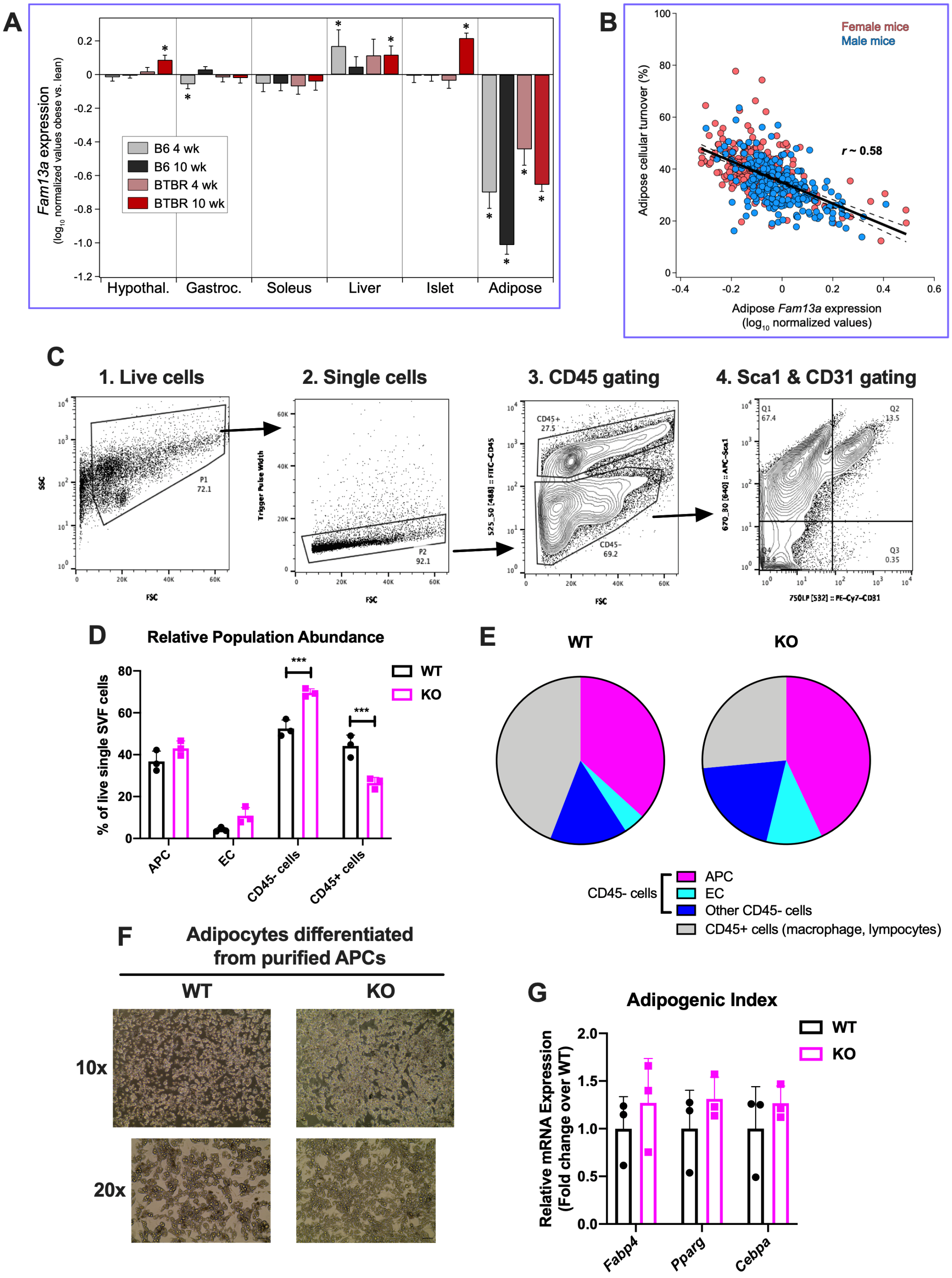

Supplementary Figure 9: Correlation of adipose F*am13a* expression with obesity in F2 cohort database. **A)** *Fam13a* gene expression in 6 key tissues (islet, liver, adipose, hypothalamus, gastrocnemius and soleus) as a function of genetic obesity (lean vs. OB/OB), parental strain (B6 vs. BTBR), and age (4 vs. 10

weeks), in mouse model of Type 2 diabetes. **B)** Negative correlation of adipose Fam13a expression with proliferation of cells in adipose tissue, measured by incorporation of deuterium into newly synthesized DNA in B6: BTBR F2 mice. **C)** Representative FACS gating strategy used to obtain adipose progenitor cells (APC) and endothelial cells (EC) in SAT of 16-week male mice fed on chow. SVFs were isolated from SAT and stained with anti-CD45 (FITC-conjugated), anti-Sca1 (APC-conjugated) and anti-CD31 (PE-Cy7-conjugated). Populations were purified as follows: 1. Cells were initially selected by size, on the basis of forward scatter (FSC) and side scatter (SSC); 2. Cells were then gated on both FSC and SSC singlets to ensure that individual cells were analyzed; 3. Cells were selected against CD45; 4. CD45- cells were then selected against Sca1 and CD31. The abundance of the relevant cell subpopulation for each mouse was provided in the Data Source (n=3 per group). **D-E**) Quantification of relative population abundance presented either in the bar chart (**D**) or in the pie chart (**E**). **F-G**) Morphological (**F**) or quantitative assessment of adipogenic degree by measuring adipogenic differentiation markers (**G**) in cells differentiated from APCs purified according to **C**). (n=3 per group, 16-week male mice on chow, scale bar=100 μm for 10x magnification, scale bar=50 μm for 20x magnification).
