# Supplementary material for: FAM13A affects body fat distribution and adipocyte function
Source: Nat Commun. 2020 Mar 19;11:1465. doi: 10.1038/s41467-020-15291-z (PMC7081215; doi:10.1038/s41467-020-15291-z)
Supplement: Supplementary file 2 — Reporting Summary [file 41467_2020_15291_MOESM2_ESM.pdf]

nature research

Corresponding author(s): Joshua Knowles
Erik Ingelsson

Last updated by author(s): Feb 19, 2020

Reporting Summary

Nature Research wishes to improve the reproducibility of the work that we publish. This form provides structure for consistency and transparency in reporting. For further information on Nature Research policies, see Authors & Referees and the Editorial Policy Checklist .

Statistics

For all statistical analyses, confirm that the following items are present in the figure legend, table legend, main text, or Methods section.

n/a	Confirmed	
☐	☒	The exact sample size (*n*) for each experimental group/condition, given as a discrete number and unit of measurement
☐	☒	A statement on whether measurements were taken from distinct samples or whether the same sample was measured repeatedly
☐	☒	The statistical test(s) used AND whether they are one- or two-sided<br>*Only common tests should be described solely by name; describe more complex techniques in the Methods section.*
☐	☒	A description of all covariates tested
☐	☒	A description of any assumptions or corrections, such as tests of normality and adjustment for multiple comparisons
☐	☒	A full description of the statistical parameters including central tendency (e.g. means) or other basic estimates (e.g. regression coefficient) AND variation (e.g. standard deviation) or associated estimates of uncertainty (e.g. confidence intervals)
☐	☒	For null hypothesis testing, the test statistic (e.g. *F*, *t*, *r*) with confidence intervals, effect sizes, degrees of freedom and *P* value noted<br>*Give P values as exact values whenever suitable.*
☐	☒	For Bayesian analysis, information on the choice of priors and Markov chain Monte Carlo settings
☒	☐	For hierarchical and complex designs, identification of the appropriate level for tests and full reporting of outcomes
☒	☐	Estimates of effect sizes (e.g. Cohen's *d*, Pearson's *r*), indicating how they were calculated

Our web collection on statistics for biologists contains articles on many of the points above.

Software and code

Policy information about availability of computer code

Data collection	Microsoft Excel is a spreadsheet developed by Microsoft for Windows, macOS, Android and iOS.
Data analysis	GraphPad Prism is a commercial scientific 2D graphing and statistics software available for both Windows and Macintosh computers.<br>Adiposoft is an automated Open Source software for the analysis of adipose tissue cellularity in histological sections. Adiposoft has been developed as a plug-in for Fiji (advanced distribution of ImageJ) that can be run under Windows, Linux or MacOS X. The software, that can be downloaded and used with no license restrictions.<br>Fiji (Fiji Is Just ImageJ) is an open source image processing package based on ImageJ.<br>ImageJ is a Java-based image processing program developed at the National Institutes of Health.<br>RNA-Seq Graphics with ggplot2.The ggplot2 package is a graphics language for creating elegant and complex plots using R.<br>R is a programming language and free software environment for statistical computing and graphics supported by the R Foundation for Statistical Computing.

For manuscripts utilizing custom algorithms or software that are central to the research but not yet described in published literature, software must be made available to editors/reviewers. We strongly encourage code deposition in a community repository (e.g. GitHub). See the Nature Research guidelines for submitting code & software for further information.

Data

Policy information about availability of data

All manuscripts must include a data availability statement. This statement should provide the following information, where applicable:

- Accession codes, unique identifiers, or web links for publicly available datasets
- A list of figures that have associated raw data
- A description of any restrictions on data availability

We have deposited the raw mouse RNA-seq data on GEO and the accession number is GSE137022. This process has been completed and the code is provided along with the publication. The public release date has been tentatively set to October 1, 2019.

Field-specific reporting

Please select the one below that is the best fit for your research. If you are not sure, read the appropriate sections before making your selection.

- [x] Life sciences
- [ ] Behavioural & social sciences
- [ ] Ecological, evolutionary & environmental sciences

For a reference copy of the document with all sections, see nature.com/documents/nr-reporting-summary-flat.pdf

Life sciences study design

All studies must disclose on these points even when the disclosure is negative.

Sample size	Sample size in animal and cell culture studies were determined using typical sample size reported in the literature. The deterministic factor for number of mice in each study/group were based on the maximum number of mice available at the colony at the time of study. For instance, in the diet studies initially we had 7 mice per each specific group. In some cases, such as body weight measurement more mice were available for instance we had 14 mice in some groups.<br>Regarding the human dataset, as those are recruited cohorts, sample sizes were based on the availability of data reported and or accessible.
Data exclusions	We have not excluded any data form our analysis.
Replication	The findings on animal studies were repeated one more time for the body weight measurement, fat depot weight in two different cohorts, and GTT and ITT in male and female mice on HFD. For example, the lower ratio of VAT/SAT in Fam13a KO mice on HFD was replicated in a second cohort. Other studies including those in cell culture, have always had at least three technical replicates.
Randomization	Studies on animal model were allocated into WT and KO mice groups with sex-specific considerations. In cell culture, studies were based on the mock and Fam13a knock-down conditions. Accordingly, we essentially did not randomize any of these conditions as the baseline measure were not different in the comparison groups.
Blinding	We blinded the slides for adipocytes size and number. Thus, the researcher who captured the images for further histomorphometry assessment was completely blind to the genotype (WT vs Fam13a KO) data.

Reporting for specific materials, systems and methods

We require information from authors about some types of materials, experimental systems and methods used in many studies. Here, indicate whether each material, system or method listed is relevant to your study. If you are not sure if a list item applies to your research, read the appropriate section before selecting a response.

Materials & experimental systems

n/a	Involved in the study	
☐	☒	Antibodies
☐	☒	Eukaryotic cell lines
☒	☐	Palaeontology
☐	☒	Animals and other organisms
☐	☒	Human research participants
☒	☐	Clinical data

Methods

n/a	Involved in the study	
☒	☐	ChIP-seq
☒	☐	Flow cytometry
☒	☐	MRI-based neuroimaging

Antibodies

Antibodies used	FAM13A (Anti-FAM13A antibody produced in rabbit, HPA038109 Sigma-Aldrich, Polyclonal, Isotype IgG)<br>CD-24 (CD24 Monoclonal Antibody (SN3b) # MA5-11833, Host / Isotype Mouse / IgM, kappa)
Validation	We validated Fam13a antibody in five tissues (lung, epididymal fat, inguinal fat, brown fat and liver) from WT versus fam13a KO mice.

Eukaryotic cell lines

Policy information about cell lines

Cell line source(s)	The specific cell line we used in this study was "Human SGBS adipocytes". The data and distribution of the cell line have already been published: Posovszky et al., Human SGBS cells - a unique tool for studies of human fat cell biology. Obes Facts. 2008;1(4):184-9.

Authentication	SGBS cells have almost unlimited source due to their ability to proliferate for up to 50 generations with retained capacity for adipogenic differentiation. So far, the cells have been used for a number of studies on adipose differentiation, adipocyte glucose uptake, lipolysis, apoptosis, regulation of expression of adipokines, and protein translocation. The cells are efficiently differentiated in the presence of PPARγ agonists and in the absence of serum and albumin (ref: Posovszky et al., Human SGBS cells - a unique tool for studies of human fat cell biology. Obes Facts. 2008;1(4):184-9.)
Mycoplasma contamination	We routinely check for Mycoplasma contamination by PCR on our frozen cells and all other cell in culture.
Commonly misidentified lines (See ICLAC register)	*Name any commonly misidentified cell lines used in the study and provide a rationale for their use.*

Animals and other organisms

Policy information about studies involving animals; ARRIVE guidelines recommended for reporting animal research

Laboratory animals	We obtained Fam13a mice, (originally created from ES cells of KOMP Repository in which exon 5 of the Fam13a gene was flanked by LoxP sites), as a contribution from Jin's lab (Department of Comparative Biosciences, University of Illinois at Urbana–Champaign, Urbana, IL). Control (WT) and KO mice were developed on C57BL6 background.
Wild animals	The study did not involve wild animals.
Field-collected samples	The study did not involve samples collected from the field.
Ethics oversight	All animal studies were reviewed and approved prior to commencement of the activity by the Administrative Panel on Laboratory Animal Care (APLAC) at Stanford University.

Note that full information on the approval of the study protocol must also be provided in the manuscript.

Human research participants

Policy information about studies involving human research participants

Population characteristics	The Human research participants were used in this study were all either published and or publicly available cohorts. The GWAS data was based on either publication and or NHLBI GRASP: Genome-Wide Repository of Associations Between SNPs and Phenotypes.<br>GRASP includes all available genetic association results from papers, their supplements and web-based content. To see detail of the meeting criteria and guidelines visit the link.<br>https://grasp.nhlbi.nih.gov/Overview.aspx<br><br>UK Biobank and Gene ATLAS<br>The UK Biobank is a prospective cohort study of approximately 502,000 individuals conducted between 2006 and 2010. The data includes genotype and detailed phenotype information; the study was approved by the North West Multi-Center Research Ethic Committee, and all participants provided written consent.<br><br>METSIM and STAGE<br>THe METabolic Syndrome In Men (METSIM) Study is a resource for studies of metabolic and cardiovascular diseases14; and The Stockholm Atherosclerosis Gene Expression (STAGE) Study, is a multi-organ expression profiling (gene network across seven vascular and metabolic tissues) to uncover a gene module in coronary artery disease gene16,42. Human gene-by-gene and gene-by trait correlations were performed on adipose tissue expression arrays (Affymetrix U219) data from the METSIM study13,43, as well as adipose and liver expression arrays (Affymetrix HG-U133 Plus 2.0) within the STAGE study16,42. These individual X gene expression matrices were used for correlations, which were performed using the WGCNA package44 in R and corrected using a 5% FDR. For the METSIM expression arrays, there were 770 individuals in total. There were 1400 biopsies taken, although only 770 subjected to the expression arrays. These were all men collected from Kupio Finland, age 45-73. The STAGE consisted of 105 individuals (both genders) collected from Sweden and Estonia, age 58-74. Here 72 individuals were subjected to expression arrays using the HuRSTA-2a520709 affy platform.
Recruitment	The Human research participants were used in this study were all either published and or publicly available cohorts.
Ethics oversight	The Human research participants were used in this study were all either published and or publicly available cohorts.

Note that full information on the approval of the study protocol must also be provided in the manuscript.
